# Word length effect in free recall of randomly assembled word lists

**DOI:** 10.3389/fncom.2014.00129

**Published:** 2014-10-14

**Authors:** Mikhail Katkov, Sandro Romani, Misha Tsodyks

**Affiliations:** ^1^Department of Neurobiology, The Weizmann Institute of ScienceRehovot, Israel; ^2^Center for Theoretical Neuroscience, Columbia UniversityNew York, NY, USA; ^3^Nizhny Novgorod Neuroscience Center, Institute of Living Systems, University of Nizhny NovgorodNizhni Novgorod, Russia

**Keywords:** long term memory, modeling, associative memory, neural network

## Abstract

In serial recall experiments, human subjects are requested to retrieve a list of words in the same order as they were presented. In a classical study, participants were reported to recall more words from study lists composed of short words compared to lists of long words, the word length effect. The world length effect was also observed in free recall experiments, where subjects can retrieve the words in any order. Here we analyzed a large dataset from free recall experiments of unrelated words, where short and long words were randomly mixed, and found a seemingly opposite effect: long words are recalled better than the short ones. We show that our recently proposed mechanism of associative retrieval can explain both these observations. Moreover, the direction of the effect depends solely on the way study lists are composed.

## Introduction

Recently we have proposed a mechanism of associative information retrieval that explicitly takes into account long-term neuronal representations of memory items (Romani et al., 2013). One of the basic predictions of the model is the existence of “easy” and “difficult” words. This prediction was verified in our analysis of a large dataset of free recall experiments collected in the lab of Michael Kahana, where we showed that the probability of words to be recalled are consistent between arbitrarily chosen groups of subjects (Katkov et al., submitted). The natural question posed by these observations is what features are predictive for the word difficulty in recall experiments, in particular what if any is the contribution of the word length.

Most of the previous studies of word length effect used lists that were specifically composed of either short or long words. In two previous studies where lists composed of alternating short and long words were used, no word length effect was observed (Hulme et al., 2004; Jalbert et al., 2011). Our current contribution uses free recall paradigm and is based on a much larger dataset than previous studies. We report that when words are selected randomly, irrespective of their length, long words are recalled *better* than short ones, in a seeming contradiction to classical word length effect in both serial and free recall (Baddeley et al., 1975; Russo and Grammatopoulou, 2003; Tehan and Tolan, 2007; Bhatarah et al., 2009). We provide a possible resolution of this contradiction in the framework of the associative retrieval model of (Romani et al., 2013).

## Materials and methods

### Experimental methods

The data reported in this manuscript were collected in the lab of M. Kahana as part the Penn Electrophysiology of Encoding and Retrieval Study (see Miller et al., 2012 for details of the experiments). Here we analyzed the results from the 141 participants (age 17–30) who completed the first phase of the experiment, consisting of seven experimental sessions. Participants were consented according the University of Pennsylvania’s IRB protocol and were compensated for their participation. Each session consisted of 16 lists of 16 words presented one at a time on a computer screen and lasted approximately 1.5 h. Each study list was followed by an immediate free recall test. Words were drawn from a pool of 1638 words. For each list, there was a 1500 ms delay before the first word appeared on the screen. Each item was on the screen for 3000 ms, followed by jittered 800–1200 ms inter-stimulus interval (uniform distribution). After the last item in the list, there was a 1200–1400 ms jittered delay, after which the participant was given 75 s to attempt to recall any of the just-presented items. All trials were used; intrusions and repetitions were removed from trials.

### The model

We assume that each word is represented by a randomly chosen population of neurons in the dedicated memory network. We further assume that each retrieved item acts as an internal cue for the next one according to similarity measure between items, which is defined as a the size of the intersection between the corresponding populations (the number of neurons that represent both items). Following (Romani et al., 2013), we consider the retrieval process that is directly determined by memory representations of the items, without explicitly simulating network activity. The dynamics of the retrieval is described by a sequence of recalled items. The first one is randomly chosen among the presented ones, and each subsequent recalled item chosen to be the one that has a maximal similarity to the currently recalled one, not counting just “visited” item (Romani et al., 2013). The recall is terminated when the retrieval process enters a cycle and no more items can be retrieved.

To mimic the experimental protocol (see above), we generated *W* = 1638 random binary patterns of length N: {${\text{ξ}}_{i}^{w}$ = 0; 1} with *w* = 1, … , *W*; *i* = 1, … , *N* indicates the neurons in the network, such that ${\text{ξ}}_{i}^{w}$ = 1 if neuron *i* is participating in the encoding of the memory item *w*. The similarity between items *w* and *w′* is then computed as ${\text{S}}^{ww^{\prime }}=\sum_{i=1}^{N}{\text{ξ}}_{i}^{w}{\text{ξ}}_{i}^{w^{\prime }}$. The pattern components for each item were drawn independently with the probability *p_w_* of ${\text{ξ}}_{i}^{w}$ = 1 chosen in the following way: each pattern was arbitrarily assigned a syllabic length *l_w_* = 1…4 such that the distribution of *l_w_* across the patterns matched the corresponding distribution across the words used in the experiment (five words with syllabic length larger than four were combined with those of length four). For patterns with given *l_w_*, corresponding *p_w_* were equidistantly distributed from 0.02 − 10^−3^*l_w_* to 0.02 + 10^−3^*l_w_*. With this choice of pattern statistics, the average number of neurons representing a given item does not depend on its syllabic length, whereas the variance is increasing with syllabic length. The word representations were then fixed throughout the simulated experiment.

For each simulated recall trial, *L* = 16 items were chosen for presentation according to two experimental protocols. For the first one, items were selected completely independently, as in the experiment of Kahana. For the second protocol, items with the same *l_w_* were randomly selected. Recall process was simulated as in (Romani et al., 2013). The first recalled item was randomly chosen among the presented ones. Subsequent transitions between recalled items were determined by the similarity matrix *S* between them, each element of which was computed as the number of neurons in the intersection between the corresponding representations: ${\text{S}}^{ww^{\prime }}=\sum_{i=1}^{N}{\text{ξ}}_{i}^{w}{\text{ξ}}_{i}^{w^{\prime }}$. More specifically, the next retrieved item is the one that has the maximal similarity to the currently retrieved one, excluding the item that was retrieved just before the current one. The recall is terminated when the retrieval process enters a cycle and no more items can be retrieved.

## Results

**Figure 1:**
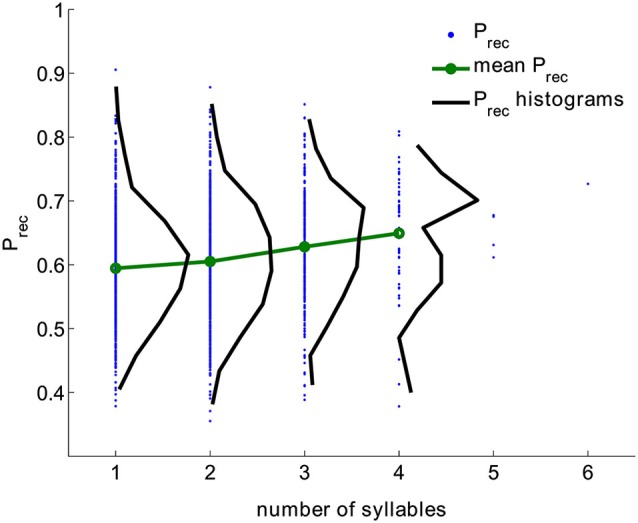
**Probability of recall for words with different number of syllables (blue dots), the distribution of probabilities of recall (black) and mean value of probability of recall (green) computed from experimental data**. Correlation coefficient between the number of syllables and recall probability is 0.15, *p* < 10^−6^).

We analyzed a large dataset of free recall experiments performed by 141 subjects with 112 trials per subject. The data is collected in the lab of Michael Kahana. The lists were composed of 16 words randomly selected from a pool of 1638 words. All trials were used; intrusions and repetitions were removed from trials (in total 15792 trials, see Section Methods). For each word, its overall recall probability (*P_rec_*) was computed as the fraction of trials this word was recalled when it was presented. Figure 1 shows the distribution of (*P_rec_*) for all words having the given number of syllables (black) aggregated from all trials. The distribution of *P_rec_* is wide for all word lengths. Nevertheless, the mean probability of recall and its variance grow monotonically with the number of syllables (correlation coefficient is 0.15, *p* < 10^−6^).

**Figure 2:**
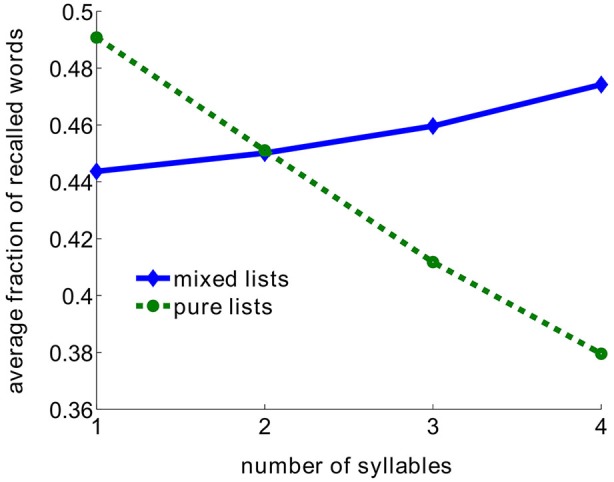
**Average fraction of recalled words as a function of number of syllables in the model**. Pure lists are composed using only words with the same number of syllables. Mixed lists are composed from the whole pool of words.

This result seemingly contradicts the classical word length effect, where lists of short words were shown to be recalled better than lists of longer words (Baddeley et al., 1975; Russo and Grammatopoulou, 2003; Tehan and Tolan, 2007; Bhatarah et al., 2009). To test whether both these effects may be explained by our proposed retrieval mechanism, we simulated the model imitating experimental paradigms in two conditions—free recall with lists composed from short/long words and random lists (see Methods). The surprising result emerged: the performance in free-recall task depends on the experimental paradigm—in recall of random mixture of unrelated words longer words are statistically easier to recall, whereas in lists composed of words with the fixed number of syllables, shorter words are easier to recall (Figure 2).

Most explanations of classical word length effects assume that total length of presented stimuli is negatively correlated with the number of recalled words. To test whether this statement is supported by the data we computed correlation between the number of syllables in presented lists and the number of recalled words. We found practically no correlation (correlation coefficient is 0.004 and is not significantly different from 0, *p* = 0.67).

## Discussion

Word length effect, i.e., the observation that lists of short words are recalled better than lists of long words (Baddeley et al., 1975) is considered to be one of the key phenomena in the theories of short-term memory (Campoy, 2011; Jalbert et al., 2011). Here we report that in free recall of unrelated words, where short and long words are randomly mixed, long words have higher recall probability than short ones, in seeming contradiction to the word length effect.

The classical word length effect is traditionally explained by either increased complexity of longer items (Neath and Nairne, 1995), or increased rehearsal time of longer items (Baddeley, 1986, 2003; Page and Norris, 1998; Burgess and Hitch, 1999). The first account suggests that shorter words are generically easier to recall, which is not compatible with our observation. In the second account, more of short words can be rehearsed, due to shorter rehearsal time, and therefore more of them are recalled. This explanation does not specify in what order one would rehearse presented words, but it suggests a negative correlation between the total length of presented items and number of recalled words, whereas no such correlation exists in data.

Here we show that our recently suggested mechanism of associative retrieval can potentially account for both the classical word length effect (which is also present in free recall experiments, see Russo and Grammatopoulou, 2003; Bhatarah et al., 2009) and the opposite length effect in lists of randomly selected words reported in this contribution. In contrast to existing models, long-term neuronal representation of items plays crucial role in our model, and no separate short-term memory mechanism is required. In particular, recall probability of items in random lists is increasing with the size of its representation relative to that of other items, and these items are recalled earlier and suppress the items with smaller representations (Romani et al., 2013). The average recall probability of the whole pool of items is however independent on the average representation size but relates negatively to the variance of representation size across the pool (Katkov et al., submitted). We therefore assumed that longer words do not, on average, have larger representation than shorter ones, but collectively have a higher variance of representation size. This assumption has currently no direct biological justification, but allowed us to reconcile the seeming contradiction between the experimental observations. In particular, it accounts for the classical word length effect, where only words with a given syllabic length are presented, and therefore the variance of representation size increases with syllabic length. In lists with mixed syllabic length, in some trials items with longer syllabic length have largest neuronal representation. When these lists are presented, longer words have larger probability to be recalled, suppressing other items from being recalled, resulting in mild positive correlation between syllabic length of an item and its recall probability.

The results presented in this contribution show that the length of the word is a prominent factor that affects the easiness for it to be recalled. However, we note that recall probabilities still show a wide distribution even for words of given length, indicating that other, yet unknown, word features also contribute to the probability to recall a word.

## Conflict of interest statement

The authors declare that the research was conducted in the absence of any commercial or financial relationships that could be construed as a potential conflict of interest.
